# Connecting Heat Tolerance and Tenderness in *Bos indicus* Influenced Cattle

**DOI:** 10.3390/ani12030220

**Published:** 2022-01-18

**Authors:** Tracy L. Scheffler

**Affiliations:** Department of Animal Sciences, University of Florida, Gainesville, FL 32611, USA; tscheffler@ufl.edu

**Keywords:** calpastatin, metabolic rate, protein degradation, thermoregulation

## Abstract

**Simple Summary:**

*Bos indicus* (also known as zebu or humped) cattle are heat tolerant and parasite resistant, which is advantageous in hot, humid climates. However, *Bos indicus* cattle are also known for excitable temperaments, slower growth, and variation in meat quality characteristics. The relationships between thermotolerance, temperament, and meat production traits are poorly understood. Due to its contribution to body weight, muscle may play an important role in determining the thermoregulatory capacity of *Bos indicus* cattle. Ultimately, defining relationships between muscle metabolism and heat tolerance are necessary in order to enhance tenderness, without sacrificing heat tolerance of *Bos indicus* breeds.

**Abstract:**

*Bos indicus* cattle are widely utilized in tropical and subtropical climates. Their heat tolerance and parasite resistance are integral for beef production in these regions; however, a reputation for excitable temperaments, slower growth, and variation in tenderness has limited their use in commercial beef production. This suggests that there is antagonism between heat tolerance and meat production traits. Meat quality characteristics are determined by the properties of skeletal muscle as well as conditions during slaughter and processing. Thus, it is possible that adaptations related to heat tolerance in the living animal affect tenderness and other meat quality attributes. Since muscle represents a large proportion of body mass, relatively small changes at the cellular level could impact overall heat production of the animal. Specifically, protein degradation and mitochondria function are aspects of organ and cellular metabolism that may help limit heat production and also have a connection to tenderness. Protein degradation postmortem is critical to structural changes that enhance tenderness whereas mitochondria may influence tenderness through their roles in energy metabolism, calcium regulation, cell death signaling, and oxidative stress. This review explores potential relationships between cellular metabolism in vivo and beef quality development in *Bos indicus* and *Bos indicus* influenced cattle.

## 1. Introduction

Animals are most productive within a range of temperatures known as the thermoneutral zone. Above or below these temperatures, the animal must use energy to maintain body temperature. High environmental temperature represents a significant challenge to livestock production due to negative effects on feed intake and performance. Projected increases in global temperatures and demand for animal protein and milk only heighten the concern for the negative impacts of heat stress on animal welfare and production. Hot climates are particularly challenging for the beef cattle industry since cattle are typically raised in outdoor settings (pasture, range, feedlot, etc.) with considerable exposure to ambient conditions. *Bos indicus* (zebu or humped) cattle exhibit enhanced thermotolerance and parasite resistance; they are often used in subtropical and tropical climates due to their ability to withstand harsh conditions. It may become increasingly necessary to incorporate *Bos indicus* cattle in beef production to impart heat tolerance. However, *Bos indicus* also possess traits that have limited their use in commercial beef production: a reputation for excitable disposition, slower growth, and less desirable carcass and beef palatability attributes. In particular, *Bos indicus* and *Bos indicus* crossbred cattle produce beef that is variable in tenderness [1,2]. Phenotypic evidence of Brahman breeding, indicated by hump height, was inversely associated with tenderness [3]. At the packing plant, hump height provides an easy means to segregate *Bos indicus* influenced cattle. In the United States, the great majority (>90%) of certified beef programs specify a limit for hump height (<2.5 inches or 6.4 cm), which is roughly equivalent to ≤25% *Bos indicus* breed composition. This criterion excludes *Bos indicus* phenotype in order to limit risk of tenderness issues. Reducing variation in beef quality and improving tenderness are important aims for increasing acceptability of *Bos indicus* cattle. However, it is important that improvements in beef quality do not negatively impact thermotolerance of *Bos indicus* cattle. 

## 2. Heat Tolerance

Compared to *Bos taurus* cattle, *Bos indicus* cattle have an improved ability to regulate body temperature in response to hot, humid environments [4,5,6]. Regulation of body temperature is a function of the heat flow between the animal and its environment, and the heat produced by metabolism. Heat exchange between the animal and its environment is dependent on surface area of the animal per unit weight, temperature between the animal and the air, and properties of the hair coat. The heat produced by the animal depends on metabolic rate. Metabolism supports maintenance of cellular functions within the animal, digestion of feed, physical activity, and production (e.g., growth or milk). Therefore, high producing animals have higher metabolic rates and energy requirements, which makes them more vulnerable to heat stress [7,8].

The superior thermoregulatory capacity of *Bos indicus* cattle appears to be a combination of increased capacity for heat loss and reduced heat production. For example, *Bos indicus* possess smooth, slick hair coats that are often light colored, which helps reflect solar radiation and prevent heat absorption by the animal. In terms of heat production, *Bos indicus* cattle appear to have decreased metabolic rates. Metabolic rate is determined by heat production of different organs and tissues of the animal. This depends on organ size as well as metabolic activity on a cellular basis. Some organs, such as the brain or liver, represent a low percentage of body weight, but exhibit high metabolic activity. On the other hand, muscle is not particularly active on a per unit basis, but it contributes significantly to metabolic rate because it represents roughly 40% of body weight. In ruminants, the liver and gastrointestinal tract contribute to >40% of heat production at rest [9]. Therefore, decreasing gastrointestinal tract and internal organ size without changing cellular metabolism could contribute to an overall reduction in metabolic rate. For steers with ≥50% Brahman composition, carcass weight represented a greater proportion of live weight (dressing percentage) compared with Angus [2]. Although carcass dressing percentage is impacted by several factors, including viscera mass and carcass fatness, this provides some evidence that size of internal organs may be a contributing factor. These data are consistent with observations that Brahman steers possess smaller livers and tend to have smaller hearts relative to body weight compared with Angus [10].

**Figure 1:**
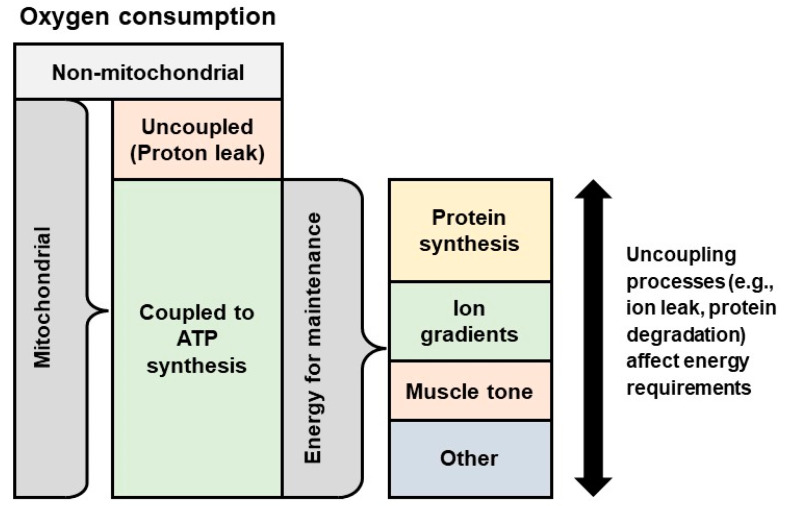
Processes related to basal metabolic rate and heat production. Oxygen consumption is an indirect measure of metabolic rate. Mitochondria are responsible for the vast majority of cellular oxygen consumption, and most oxygen consumption by mitochondria is coupled to ATP synthesis. Energy is used to support protein synthesis, maintain ion gradients, and perform other cellular maintenance. Decreasing uncoupling processes, including proton leak in mitochondria, ion leak, and protein degradation, would thus restrain metabolic rate and reduce heat production. Adapted from [11].

On a cellular level, nutrients from the diet are metabolized to accomplish vital functions, including protein synthesis, muscular contraction, and the maintenance of ion gradients across membranes. However, the energy demand does not come from these processes; rather the additional energy required for cellular maintenance is due to processes that oppose these functions [11]. These opposing or uncoupling processes include protein turnover, muscle relaxation, and ion leaks. Consequently, decreasing any of these uncoupling processes would be expected to decrease the animal’s metabolic rate and energy requirements (Figure 1). Accordingly, the decrease in metabolic rate would dictate the extent to which uncoupling processes are reduced and the organs that are impacted. 

Decreasing metabolic rate at the cellular level ultimately impacts whole animal basal metabolic rate. Consistent with this, Nellore bulls had lower net energy requirements for maintenance compared with Angus bulls [12]. Further, in a population composed of Angus, Brahman, and Angus × Brahman crossbred calves, the calves with greater fractions of Brahman breeding used feed more efficiently than calves that were predominantly Angus [13]. In the latter study, Brahman calves also consumed less feed, which decreases heat production from digestion. Reduced intake also limits energy available for production and is consistent with lower growth rate (or lower average daily gain). Together, these observations indicate lower metabolic rates in *Bos indicus*, which may be the result of a smaller size of metabolically active organs, reduced uncoupling processes at the cellular level, or a combination.

## 3. Beef Tenderness

Beef tenderness is a complex trait influenced by inherent muscle properties, as well as conditions that exist in muscle after slaughter and during processing. Major factors that affect tenderness include connective tissue [14], marbling or intramuscular fat [15], and postmortem protein degradation [16]. Age of animal and location of the meat cut explain a large fraction of connective tissue related variation in beef tenderness. Conversely, if the same cut from various carcasses within an age or maturity group is evaluated, marbling and protein degradation are the main factors associated with palatability. While marbling is a challenge for *Bos indicus* cattle, it only has a small, positive effect on palatability [17]. Greater toughness is largely attributed to decreased activity of protein degradation systems postmortem. During the refrigerated storage of meat (aging), endogenous proteolytic enzymes disrupt the structural integrity of muscle, which contributes to tenderization. Muscle characteristics and postmortem factors affect the rate and extent of proteolysis. 

Considering muscle represents a large proportion of body weight, muscle characteristics could be an important determinant of overall body metabolism and heat tolerance. In turn, the adaptations in muscle of *Bos indicus* cattle may impact beef tenderness. Reduced protein degradation in muscle has attracted interest because it would help explain not only reduced growth rates and metabolic rates in *Bos indicus*, but also tougher beef. Muscle growth is an energetically demanding process that contributes to metabolic heat production. In order to increase muscle mass, proteins must be synthesized as well as degraded. The net balance of synthesis and degradation dictates accretion, or the gain in muscle mass. In living muscle, several systems contribute to protein degradation: the ubiquitin proteasome, calpain-calpastatin, cathepsins, and caspase systems. Of these, the major player in postmortem muscle is the calpain-calpastatin system [18,19]. Calpain cuts proteins into fragments, which disrupts the structure of muscle cells and contributes to the tenderization of beef. Calpastatin, on the other hand, is the only known inhibitor of calpain [20]. *Bos indicus* cattle are well-documented to possess elevated calpastatin activity in postmortem muscle, consequently decreasing degradation and limiting tenderization [21,22,23]. Therefore, greater calpastatin observed in muscle of *Bos indicus* breeds may be a mechanism for limiting protein turnover in the animal in order to restrict metabolic heat production; in postmortem muscle, this manifests as tougher beef.

### 3.1. Calpains and Calpastatin

The calpains are a group of calcium dependent cysteine proteases located ubiquitously within the cell (reviewed by [20]). There are fourteen members in the calpain family; however, only µ-calpain and m-calpain (also known as calpain 1 and calpain 2, respectively) are implicated in postmortem protein degradation and meat tenderness. Micro- and milli- refer to the concentrations of calcium required for their activation. In the presence of calcium, both µ- and m-calpain autolyze; the 80 kDa unit is reduced to a 78 kDa intermediate, followed by a 76 kDa product. Autolysis is considered an indicator that calpains have become proteolytically active, however the absence of autolysis does not mean that calpains are inactive [24,25]. Once calpains autolyze, the calcium needed for proteolytic activity decreases. Calcium concentrations needed for µ-calpain decrease from 30–50 µM to 0.5–2.0 µM for half maximal activity, and concentrations for m-calpain decrease from 400–800 µM to 50–150 µM [20]. Calpastatin is the endogenous inhibitor specific to calpains. Currently there is no evidence that calpastatin inhibits any other proteases. Calpastatin has four domains that can each inhibit the proteolytic activity of calpains. Calpastatin is heat stable and labile to proteolytic degradation, but calpastatin fragments retain inhibitory activity.

Calpains have a broad variety of substrates, including key structural and contractile proteins within muscle cells. Incubating myofibrils with calpains produces similar patterns in protein degradation as those observed in postmortem muscle; myofibrillar proteins such as desmin, troponin-T, titin, and nebulin are cleaved in meat during aging [26]. Calpain also cleaves calpastatin, and the ratio of calpastatin: µ-calpain activity is considered a good predictor of tenderness. Tenderization rates between species (beef < lamb < pork) are inversely associated with calpastatin: µ-calpain [27]. The calpastatin: µ-calpain activity ratio is also elevated in callipyge lambs, which produce meat with reduced proteolysis and higher shear force than control lambs [28]. The calpastatin: µ-calpain ratio is also generally less favorable in *Bos indicus* cattle. Muscles from *Bos indicus* cattle exhibit greater calpastatin activity than *Bos taurus*, and as Brahman influence increases, the calpastatin: µ-calpain ratio increases [29]. *Bos taurus* and *Bos indicus* cattle exhibit differences in muscle calpastatin activity both pre-rigor (45 min postmortem) and post-rigor (48 h) [29]. This is consistent with the idea that differences in calpastatin inhibitory activity exist in living muscle. Thus, reducing protein degradation in living muscle would be expected to enhance muscle growth, as long as protein synthesis rates are maintained or increased. For instance, callipyge lambs are well-known for extreme muscle hypertrophy that manifests postnatally; the much higher calpastatin activity in longissimus is evidence for reduced protein degradation that would ultimately enhance protein deposition and muscle growth [28]. Along these lines, the growth promoting effects of anabolic implants and β-adrenergic agonists may be partly mediated by relatively small decreases in protein degradation [30,31]. However, greater calpastatin: µ-calpain activity in *Bos indicus* is not linked to enhanced muscle growth. In the case of *Bos indicus*, decreased protein degradation may be accompanied by lower rates of protein synthesis, resulting in similar or slower rates of growth compared with *Bos taurus*. 

Due to their key roles in tenderization, µ-calpain and calpastatin are important targets for understanding the variation in tenderness in *Bos indicus* beef. Even though the association between calpastatin activity and tenderness is well documented, the underlying mechanisms remain poorly understood. Several single nucleotide polymorphisms in µ-calpain and calpastatin have been associated with tenderness but the relationships are inconsistent and depend on the population evaluated, and in many cases, the polymorphisms are not functional mutations [32,33,34,35]. Calpastatin is particularly complex, and its regulation remains poorly understood. The calpastatin gene contains multiple promoters, resulting in several different transcripts that may be alternatively spliced into mRNAs [36]. A variety of calpastatin isoforms have been identified in various tissues [20]. Calpastatin isoforms migrate more slowly than expected in sodium dodecyl sulfate polyacrylamide gel electrophoresis. In skeletal muscle, the predominant forms are typically observed between 115 and 135 kDa, which is much larger than predicted based on the amino acid sequence. Moreover, since proteases cleave calpastatin, it is not always clear if the multiple bands are distinct isoforms or the result of cleavage. In the case of postmortem muscle, the aforementioned large band is cleaved and becomes weaker with time.

Greater content of calpastatin protein may partly explain increased calpastatin activity and toughness. Polymorphisms in calpastatin identified in an Angus-Brahman multibreed population may affect tenderness by changing mRNA stability, which would affect expression of calpastatin protein [34]. Relative to Angus, Brahman longissimus has been shown to contain greater calpastatin protein. In a small study with Nellore and Angus bulls, calpastatin activity was a better predictor of tenderness than protein expression [37]. While calpastatin expression was numerically higher in Nellore, this also suggests that additional mechanisms may be involved in regulating calpastatin activity. In other cell types, phosphorylation is a means of regulating its cellular distribution and localization, thereby affecting its ability to inhibit calpain [38,39]. Along these lines, two distinct pools of calpastatin in muscle can be separated using anion exchange chromatography, with the second fraction contributing greater inhibitory activity [24]. During aging, the decline in calpastatin activity in both the triceps brachii and longissimus lumborum was primarily due to a decrease in the second fraction of calpastatin [40], which was previously demonstrated to be a phosphorylated form [38]. 

### 3.2. Muscle Properties of Bos Indicus versus Bos Taurus

Muscle is a heterogeneous tissue, and the properties of individual cells (fibers) vary to meet specific functions. Moreover, fiber properties adapt to changes in physical activity, hormonal influences, or other environmental stimuli. Since protein turnover rate is associated with muscle properties, muscle characteristics may contribute to variation between biological types. Muscle fibers are classified according to their contractile (slow vs. fast) and metabolic properties (oxidative vs. glycolytic), which are largely dictated by function. Slow contracting fibers rely primarily on oxidative metabolism, making them well-suited for endurance activities, whereas fast-twitch glycolytic muscles are designed for short, intense bursts of activity. The contractile speed is largely determined by myosin heavy chain isoform expression. In cattle, fibers express type I (slow), IIa or IIx (fast) myosin heavy chain isoforms. For living muscle, fiber recruitment during activity may dictate protein turnover. The motor units of type I and IIa fibers are generally activated more frequently, and higher rates of protein synthesis may be required to match higher use dependent degradation [41]. However, contractile fiber type is not likely to be related to tenderness and proteolysis differences between *Bos taurus* and *Bos indicus* as there are several reports that the subspecies exhibit similar protein expression of myosin heavy chain isoforms [22,42].

Alternatively, metabolic characteristics and signaling pathway mediated control of metabolism may be more important targets for investigation in *Bos indicus* muscle. Based on their reputation for excitable temperaments, the stress response has been proposed to underlie variation in growth and tenderness. Steers designated as excitable based on flight speed and chute score exhibit higher blood cortisol, and lower growth performance and feed efficiency [43,44] and decreased beef quality, indicated by lower redness and higher shear force [45,46]. Cortisol is more often associated with long term stress whereas acute stress preslaughter triggers catecholamine release. Stimulation of β-adrenergic receptors in muscle has been shown to increase calpastatin expression [47]. Thus far, there is little direct evidence for temperament impacting calpastatin activity [48,49]. Evaluating the role of temperament in tenderness is complicated by different duration and nature of stressors and their effects on other aspects of muscle metabolism, which influence meat quality development.

### 3.3. The Conversion of Muscle to Meat

**Figure 2:**
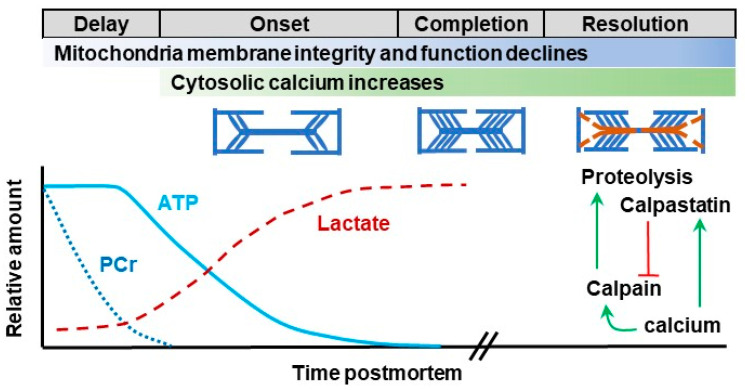
Biochemical, physical, and energetic changes during the conversion of muscle to meat. During the delay phase of rigor mortis, phosphocreatine (PCr) helps maintain ATP levels; as PCr supply diminishes, anaerobic conversion of glycogen to lactate becomes the primary means of ATP production. As ATP becomes limiting, permanent actomyosin crossbridges form that increase muscle tension (onset). Tension is maximal when ATP is exhausted (completion). Subsequently, muscle tension decreases (resolution) due to degradation of proteins by endogenous enzymes. The calcium activated calpain/calpastatin system plays a significant role in disruption of structural proteins, resulting in tenderization. Mitochondria are proposed to participate in these changes through their roles in energy metabolism, calcium regulation, cell death, and oxidative stress.

At slaughter, muscle does not immediately become meat; rather, many changes occur during the “conversion of muscle to meat.” The physical, biochemical, and energetic changes that ensue in muscle after slaughter are critical for determining the development of meat quality attributes, such as tenderness (Figure 2). Exsanguination results in a loss of blood supply, thereby eliminating oxygen delivery and waste removal. However, muscle attempts to maintain homeostasis and uses energy (ATP) for muscle relaxation and calcium sequestration. The limited oxygen supply leads to a shift in metabolism towards anaerobic pathways for energy production. Initially, phosphocreatine is used to generate ATP. Once 70% of phosphocreatine pool has been used, ATP levels decline relatively quickly [50]. Muscle glycogen is metabolized through anaerobic glycolysis in order to generate ATP, which also produces lactate and H^+^. The accumulation of H^+^ lowers muscle pH. As the breakdown of ATP exceeds its production, less ATP is available and permanent actomyosin crossbridges form, leading to an increase in muscle tension and shortening of sarcomeres. Once ATP is completely exhausted, the actomyosin crossbridges cannot be broken and rigor mortis (“stiffness of death”) is complete. At this point, the muscle is relatively inextensible and tension is maximal. Along with the physical changes, the pH of bovine muscle will decrease from around pH 7.2 (living muscle) to roughly 5.5 to 5.8. During this time, carcasses will also cool from body temperature (38.5 °C) to <4 °C at 24 h postmortem. 

The muscle tension from rigor mortis decreases during postmortem storage as a result of degradation of myofibrillar proteins and loss of structural integrity. As indicated earlier, endogenous enzymes in muscle contribute to postmortem proteolysis and tenderization. However, shifts in temperature, pH, energy status and ionic strength affect enzymatic activity and thus alter the capacity for the aforementioned protease systems to contribute to protein degradation. Although refrigerated storage of meat for several weeks after slaughter (aging) improves beef tenderness, the majority of tenderization caused by protein degradation occurs in the first 24–72 h postmortem. In fact, approximately 50% of the structural and myofibrillar changes occur within the first 24 h postmortem [18]. During the early postmortem period, changes in pH, temperature, and sarcoplasmic calcium concentration affect activation of calpain. However, calpastatin and µ-calpain rapidly lose activity postmortem, whereas m-calpain is less affected by longer aging periods [51]. For instance, native m-calpain activity decreased with longer aging times while autolyzed m-calpain activity increased in longissimus aged up to 42 day [52]. In total, µ-calpain and calpastatin likely drive tenderization during the first several days postmortem, while m-calpain may contribute to tenderization during prolonged aging.

The rate and extent of changes in muscle pH and temperature early postmortem are important factors associated with µ-calpain activity. In vitro analysis revealed that µ-calpain activity is higher at pH 6.5 compared to pH 6.0 and 7.5, and higher ionic strength decreased calpain activity regardless of pH [53], suggesting that a faster rate of pH decline may favor autolysis and activation of calpain. However, an extremely rapid rate of pH decline with high muscle temperature is detrimental to µ-calpain activity and aging potential [54,55]. In bovine longissimus, the ultimate pH (pH_u_) is also linked to variation in tenderness. High pH_u_ (pH ≥ 6.2) was associated with rapid autolysis of calpains and more rapid degradation of titin, nebulin, and filamin, while a low pH_u_ (pH ≤ 5.79) caused a more rapid degradation of desmin [25]. With these contrasting pH_u_ values, it is also expected that the muscle temperatures at a given pH would differ, which would contribute to variation in enzymatic activity and proteolysis. A minimal degree of shortening occurs when muscle is allowed to go into rigor at 15–20 °C [56]. Warmer temperatures typically favor greater enzymatic activity, and the lesser degree of shortening would maintain spacing and potentially increase the ability of µ-calpain to access target proteins. In a model using in vitro digestion of purified myofibrils, temperature (22 vs. 4 °C) had a greater impact on µ-calpain activity than the rate of pH decline [57]. Similarly, intermediate temperatures (between 10 and 25 °C) may favor optimal calpain activity [54]. In total, there is an ideal rate of temperature and pH decline that favors activation of µ-calpain and subsequently prolongs the potential for proteolysis and tenderization during the aging period. 

Stressors may indirectly affect proteolysis through effects on pH decline and postmortem metabolism. Long-term stress contributes to glycogen depletion in muscle, subsequently limiting the rate and extent of pH decline. In more extreme cases, this results in “dark cutters,” which are characterized by firm, dry surface texture and dark purplish-red lean. Conversely, epinephrine promotes signaling pathways that stimulate glycogen degradation and hasten the rate of postmortem glycolysis and pH decline. Though different mechanistically, these pathways alter pH decline, and therefore can affect calpain activity and proteolysis.

### 3.4. Mitochondria

Though there has been significant progress in understanding changes in postmortem muscle, there is still much that is poorly understood. The contribution of mitochondria has long been ignored, largely because the removal of the oxygen supply would negate their capacity to contribute to ATP production. Yet, there is oxygen remaining in muscle at harvest, and mitochondria may use this oxygen for ATP production if they are functionally competent. For instance, electrical stimulation of bovine longissimus hastens the decline in muscle oxygenation, and this is associated with more rapid glycolysis and pH decline and compromised mitochondrial function [58]. Isolated mitochondria retain the capacity for ATP production for some time postmortem, and we have also found that mitochondria in permeabilized fibers are well-coupled and intact at 1 h postmortem [59,60].

In living muscle, mitochondria function contributes to adaptation and whole animal thermal tolerance [61]. Specifically, tighter coupling between mitochondria proton pumping and ATP synthesis enhances mitochondrial efficiency; more energy is produced and less heat is dissipated. In the case of postmortem muscle, this could enhance capacity for ATP production and affect the course of pH decline. Permeabilized fibers from longissimus collected at 1 h postmortem showed greater efficiency of oxidative phosphorylation, indicated by greater coupling, compared with Angus [59]. In conjunction, Brahman longissimus also exhibited greater resistance to pH decline compared with Angus, and calpain autolysis and protein degradation were also delayed in Brahman [62]. In muscles with divergent metabolic and contractile traits, the glycolytic longissimus exhibited a more rapid loss in outer mitochondrial membrane integrity and oxidative phosphorylation than the oxidative diaphragm [60]. Importantly, using permeabilized fibers allows for analysis of all mitochondria in the sample, regardless of their functional state. The contrasting patterns in mitochondria function in longissimus and diaphragm may be related to distinct calcium regulation between fiber types. The ability and capacity for mitochondria to sequester calcium influences the amplitude and duration of calcium oscillation within the sarcoplasm. Importantly, elevated calcium in the sarcoplasm would promote calpain-mediated proteolysis. Along these lines, inhibition of mitochondrial calcium transport enhanced free calcium in the sarcoplasm, postmortem proteolysis, and tenderization in bovine longissimus [63].

Mitochondria have also been implicated in tenderization through their role in caspase activation. In pathological situations, mitochondrial dysfunction can lead to the release of cytochrome c from the intermembrane space of mitochondria. In turn, this activates initiator caspases which subsequently activate executioner caspases, including caspases 3, 6, and 7. The caspases are responsible for proteolysis associated with programmed cell death. Caspase-3 has received attention for tenderness since it can target calpastatin [64]; in this way, caspase-3 would indirectly promote calpain activity by reducing calpastatin-mediated inhibition of calpain. There has been some evidence suggesting a role for caspase-3 in tenderization [65,66]. However, procaspase-3 must be cleaved for activation [67], and there is minimal evidence that caspase-3 cleavage occurs early postmortem [62,68,69]. Thus, calpain and calpastatin remain as the dominant players in postmortem proteolysis. 

Mitochondria represent the primary site for reactive oxygen species production in the cell. As with calcium, small amounts of reactive oxygen species can serve as signaling molecules, while large amounts can lead to oxidative stress, mitochondria dysfunction, and cell death. Oxidation affects protein function, and this may be an important mechanism influencing µ-calpain activity. Similarly, oxidation of target proteins may influence their susceptibility to degradation by proteases [70,71]. Altogether, these data support several potential direct and indirect mitochondrial contributions to postmortem metabolism. However, given the changing cellular environment early postmortem and heterogeneous nature of skeletal muscle, more work is necessary to fully understand metabolic changes that occur and their impact on proteolysis and tenderness.

## 4. Conclusions

*Bos indicus* cattle impart heat tolerance that is critical for production in hot, humid climates. However, they also have a reputation for slow growth and variation in tenderness. Certainly, there is an economic incentive to increase growth performance and improve product consistency. Because muscle represents a significant portion of body weight, metabolic properties of muscle may contribute to heat production and thus affect the thermoregulatory capacity of *Bos indicus* cattle. On a cellular level, there are several possible connections between heat tolerance and tenderness, including but not limited to, calpastatin, mitochondrial function, and stress response. Defining the relationships between muscle characteristics and heat tolerance are critical to developing strategies that optimize growth and tenderness without sacrificing the heat tolerance of *Bos indicus* breeds. 

## Data Availability

No new data were created or analyzed in this study. Data sharing is not applicable to this article.
